# Catching a New Zeolite as a Transition Material during Deconstruction

**DOI:** 10.1021/jacs.3c00423

**Published:** 2023-04-11

**Authors:** Qiudi Yue, Gwladys Steciuk, Michal Mazur, Jin Zhang, Oleg Petrov, Mariya Shamzhy, Mingxiu Liu, Lukáš Palatinus, Jiří Čejka, Maksym Opanasenko

**Affiliations:** † Department of Physical and Macromolecular Chemistry, Faculty of Science, Charles University, Hlavova 8, Prague 2 128 43, Czech Republic; ‡ Institute of Physics, Academy of Sciences of the Czech Republic, v.v.i., Na Slovance 2, Prague 8 182 21, Czech Republic; § Department of Low-Temperature Physics, Faculty of Mathematics and Physics, Charles University, V Holešovičkách 2, Prague 8 180 00, Czech Republic

## Abstract

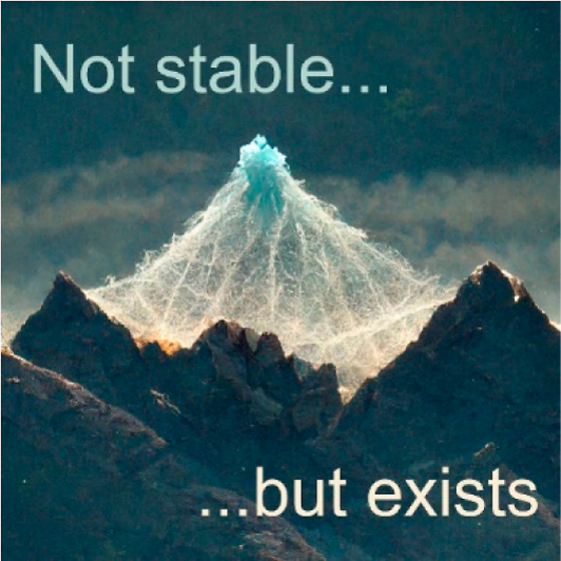

Zeolites are key materials in both basic research and industrial applications. However, their synthesis is neither diverse nor applicable to labile frameworks because classical procedures require harsh hydrothermal conditions, whereas post-synthesis methods are limited to a few suitable parent materials. Remaining frameworks can fail due to amorphization, dissolution, and other decomposition processes. Nevertheless, stopping degradation at intermediate structures could yield new zeolites. Here, by optimizing the design and synthesis parameters of the parent zeolite IWV, we “caught” a new, highly crystalline, and siliceous zeolite during its degradation. IWV seed-assisted crystallization followed by gentle transformation into the water–alcohol system yielded the highly crystalline daughter zeolite IPC-20, whose structure was solved by precession-assisted three-dimensional electron diffraction. Without additional requirements, as in conventional (direct or post-synthesis) strategies, our approach may be applied to any chemically labile material with a staged structure.

## 1 Introduction

Zeolites are crystalline silica-based materials with branched micropore systems.^1^ Thanks to their micropores, these materials can incorporate appropriately sized guest molecules, highlighting their potential as open and low-density frameworks. These open frameworks are, however, thermodynamically less stable than dense materials with similar compositions, such as quartz and dense silicates.^2−4^ Yet, despite their thermodynamic instability, some zeolites are known as core materials for oil refining and petrochemistry, attesting their stability under harsh reaction conditions.

As metastable materials resulting from synthesis iterations, zeolites are produced via hydrothermal approaches at temperatures of 100–250 °C and pressures of 1–20 bar.^5^ These conditions reflect a compromise between the formation rate of the desired zeolite phase and the rate of its subsequent structural transformation. The latter usually leads to crystal structure densification at decreasing framework energy,^6,7^ but the low framework density is not necessarily related to a high framework energy.^8^

**Figure 1:**
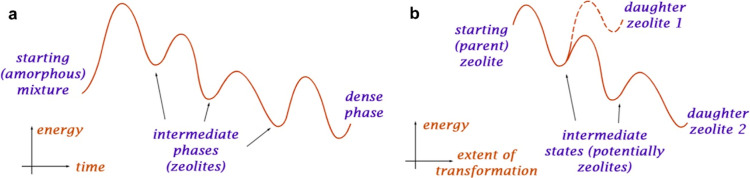
Schematic representation of the conceptually similar evolution of free energy states in zeolite synthesis. (a) Hydrothermal zeolite synthesis and (b) ADOR transformation.

The transformation of the initial amorphous phase, followed by the temporary formation and subsequent conversion of less stable zeolite intermediates into more stable products, possibly including other zeolites, is a continuous process, as schematically illustrated in Figure 1a. This continuous process explains the range of zeolites formed during their synthesis.^9^ The fairly fast crystallization of the targeted phase along with the relatively slow formation of other phases explains the wide range of specific zeolites on the synthesis timeline.

During classical zeolite synthesis, the transition from one framework to another is determined by the decrease in the Gibbs free energy of the resulting lattice.^10^ The variation in the entropy component favors crystallization when intermediate stages have a similar energy or minimal structural alterations. This experimentally observed trend is expressed in Ostwald’s step rule,^11^ according to which “the phase with the lower kinetic barrier forms first” even if less stable.

From this perspective, the multi-step assembly–disassembly–organization–reassembly (ADOR) method^12^ can be considered an analogue of hydrothermal synthesis. ADOR also proceeds through relatively stable crystalline intermediates and intermediate phases structurally resembling each other and having a gradually lower free energy. As in classical synthesis, a less feasible optional framework can be stabilized, e.g., using an organic structure-directing agent (SDA) or changing the surface charge.^13,14^ ADOR is applied to germanosilicate zeolites consisting of stable Si-rich layers connected by double-four-ring units (D4Rs) predominantly occupied by Ge atoms (>4 Ge atoms of the 8 atoms in D4R).^15^ This strategy consists of selectively transforming D4R units while preserving structural fragments, such as dense or porous layers, thereby producing daughter zeolites.^12^

Rather than yielding daughter zeolite phases, ADOR may alternatively lead to framework decomposition, yielding either amorphous or dense crystalline phases (e.g., GeO_2_) more thermodynamically stable than their parent zeolites. The lability of parent zeolites is derived from their chemical compositions because only materials with enough Ge atoms in the lattice are suitable for ADOR.^16^ Yet, whether toward novel zeolites or dense phases, the ADOR pathway always contains local minima of free energy (Figure 1b). Appropriately combined treatment factors result in a single phase with high crystallinity in which the local minima of free energy correspond to new zeolites.^13^

In this study, we postulated that complementary to the classical ADOR, novel zeolites could be “caught” during the post-synthesis transformation of a parent zeolite (germanosilicate) not leading to the stable daughter zeolite as the final material. Thus, we targeted structures formed as intermediates due to the rearrangements and/or disintegration, leading to amorphization of the parent material at the final step. Using this approach, we “caught” a daughter phase related to IWV zeolite, which was (at first glance) unsuccessfully subjected to the previously described ADOR method. By analyzing key factors influencing the lifetime of the new intermediate phase, we optimized the synthesis and post-synthesis treatment parameters, ultimately isolating a single phase of the new zeolite IPC-20.

## 2 Results and Discussion

IWV contains Ge-rich D4R units susceptible to structural modification via ADOR. However, as with many other appropriate parent zeolites, IWV has not yet been transformed into a novel daughter zeolite. Assuming that the lifetime of the potential new phase can be maximized by tuning intrinsic characteristics of IWV and conditions for its transformation, we focused on designing the starting zeolite and controlling reaction rates.

On the one hand, we tuned the chemical composition and crystal morphology of the starting zeolite for selective and complete disassembly while avoiding undesirable events, such as (i) degradation of non-D4R building units, which may be caused by a high fraction of Ge occupying intralayer crystallographic positions, and (ii) recrystallization toward the parent zeolite if the Ge content in D4Rs is too low or if severe crystal intergrowth occurs upon crystallization of the parent IWV. On the other hand, we balanced the rates of the initial disassembly and subsequent consumption of the precursor of the targeted phase (later identified as IPC-20) to lengthen its lifetime.

### 2.1 Optimization of the Chemical Composition and Crystal Morphology of the Parent IWV Zeolite

IWV was originally synthesized as an aluminosilicate zeolite using a phosphorus-containing SDA in fluoride medium.^17^ But since nitrogen-derived SDAs were found to enable the formation of aluminosilicate IWV,^18^ the range of possible IWV compositions has been since further expanded to germanosilicates,^19^ rendering this zeolite suitable for ADOR. Despite containing most Ge atoms in D4R units, these IWV germanosilicates showed a relatively high Si/Ge molar ratio of 9.8, thus accounting for the abundance of [5 Si, 3 Ge] and [7 Si, 1 Ge] in D4Rs.^19^ According to its mechanism,^13^ an ADOR structural transformation requires more than 4 Ge atoms per D4R unit. Therefore, assuming that Ge atoms exclusively occupy T-atom positions in D4R units of IWV, we should be able to prepare the corresponding ADOR-susceptible parent zeolite with a Si/Ge ratio lower than 8.5.

To increase the concentration of Ge above the level offered by the reported synthesis protocol,^19^ we first performed the synthesis under the same conditions, albeit decreasing the Si/Ge ratio in the reaction mixture. Crystallization of gels with Si/Ge = 1, 2, and 3 produced pure phases of Ge-enriched IWV materials (Figure S1) with Si/Ge = 6.6, 7.2, and 9.8, respectively. These parent zeolites had the appropriate chemical composition (Si/Ge < 8.5), but the resulting IWV-*n* (*n* stands for the Si/Ge ratio in the synthesis gel) materials did not undergo structural transformation when subjected to acidic treatments according to ADOR protocols^20^ (Figure S2). Their resistance to structural transformation was explained by their morphology, with a high degree of crystal intergrowth in large, peanut-shaped agglomerates of plate crystals (80 × 30 × 15 μm), as shown by SEM (Figure S3). Within such heavily intergrown particles, neighboring layers in the IWV zeolite framework were fixed to each other through twinning crystallites, preventing the required shrinkage of the interlayer space during disassembly and thus all further ADOR steps.

To optimize the crystal morphology of IWV, we applied the seeding method^21^ while maintaining the remaining synthesis parameters unchanged. Adding 2 wt % of IWV-3 crystals as seeds into the synthesis mixtures with Si/Ge ≤ 2 enabled us to acquire a single phase of high-quality IWV zeolites (*V*_micro_ = 0.216–0.220 cm^3^ g^–1^ (Figure S4) vs 0.18 cm^3^ g^–1^ reported in the literature^19^), with a significantly reduced degree of aggregation, as shown by SEM in Figure S5. There still was a minor fraction of intergrown IWV particles in samples obtained using the seeding approach, although these species were not so heavily integrated (Figure S5) as IWV crystals synthesized by the conventional method (Figure S3). Seeding generated uniform and predominantly independent platelet crystals, which were smaller (10 × 2 × 0.2 μm) than those synthesized otherwise. The zeolite yield also increased to 50–60% when using the seeding method, in contrast to the yields without seeding, which were lower than 15%. The Si/Ge ratios of samples prepared according to this optimized procedure from reaction mixtures with Si/Ge = 1 and 2 were 4.3 and 5.2, respectively. Assuming the preferential location of Ge in D4R units,^22,23^ the fraction of Ge atoms occupying the framework positions in these materials should suffice for subsequent disassembly via ADOR.^13^

Despite their change in chemical composition, IWV samples prepared from reaction mixtures with different Si/Ge ratios showed no change in their distribution of Si or Ge atoms between different crystallographic positions. ^29^Si and ^19^F MAS NMR spectra of IWV materials with Si/Ge ranging from 4 to 10 were similar, thus confirming that these frameworks had comparable surroundings of Si atoms and F^–^ ions post-synthetically incorporated into D4R units, respectively (Figure S6). In particular, ^19^F MAS NMR spectra predominantly displayed peaks related to Ge-rich D4Rs,^24,25^ albeit without apparent differences between samples, explaining their different behaviors upon subsequent treatments.

### 2.2 Adjustment of the Rates of Individual Steps during Structural Transformations

Acid concentration and temperature are the key parameters that determine the relative rates of ADOR transformation steps under various conditions.^26^ To specify the conditions under which new zeolite phases could exist, diluted (0.1 M) or concentrated (12 M) acid solutions were applied at low (25 °C) or high (100 °C) temperatures as limiting conditions for kinetic studies. Thus, the optimal basic parameters of hydrolysis were identified to specify the conditions that lengthened as much as possible the period when a desired intermediate zeolite phase is stable and highly crystalline.

Tracking shifts in the positions and intensities of hkl diffraction peaks in XRD patterns is the simplest way to follow changes in a zeolite structure after a treatment. “Interlayer” h00 peaks are good representatives as they describe changes in layer spacing. The absolute values of shifts in h00 peak positions directly reflect changes in interlayer distance, that is, the higher the 2θ value is, the shorter the gap will be. Interlayer distance decreases due to the disintegration of D4Rs (cubic units formed by 8 T atoms), which form S4R units (square, 4 T atoms) or regular Si_layer_–OH silanol defects between layers. Condensation of the latter results in the formation of Si_layer_–O–Si_layer_ oxygen bridges (0 T atoms in the interlayer connection). If the interlayer units (D4R, S4R, and *–O–*) are only present in the lattice or uniformly alternate in the structure (e.g., as a sequence ...ABABA...), the corresponding material is considered ordered and hence a target.

**Figure 2:**
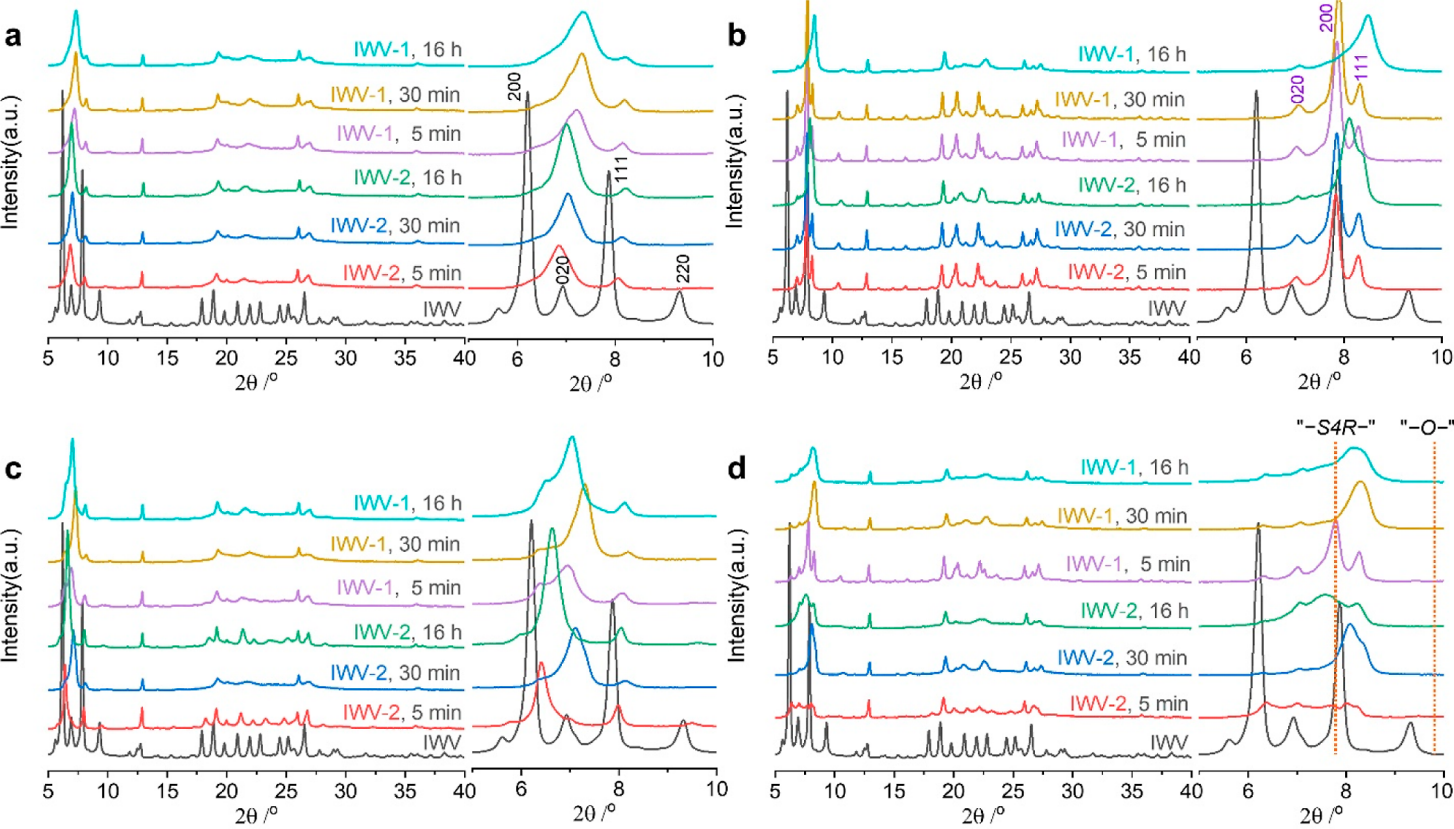
Evolution of the IWV structure during transformation under acidic conditions. XRD patterns of IWV-1 and IWV-2 samples treated with 0.1 M HCl for different times (a) and subsequently calcined at 450 °C (b). XRD patterns of IWV-1 and IWV-2 samples treated with 12 M HCl for different times (c) and subsequently calcined at 450 °C (d). The images in the right part of each section show an enlarged segment of the corresponding diffraction pattern in the range of 2θ 5–10°. Two lines in (d) represent the positions of the (200) reflection estimated for IWV-derived daughter zeolites containing different linkers, “–S4R–” or “–O–”, between the layers. For simplicity, the nomenclature (in terms of crystallographic axes) for indexing the diffraction lines in daughter IPC-20 was kept the same as for the parent IWV, although the conversion of crystallographic orientations in IPC-20 occurs (vide infra).

The position of 200 diffraction peak in potentially ordered materials derived from IWV can be predicted based on the type of connecting units. Their values (dotted lines for the S4R and *–*O*–* connections, Figure 2) must be matched to obtain the desired phase with uniform interlayer units, but the same peak position can be reached for other reasons (e.g., a combination of connecting units in the physical mixture of derivative materials). Another type of non-targeted material containing layers with poor crystallinity is specified by the decrease in the intensities of “intralayer” 0kl peaks and by the shift in their positions.

Under all conditions applied in the IWV treatment, the 200 diffraction peak was shifted to higher angles (Figure 2). The presumably ordered phase (IPC-20) with −S4R-interlayer units is formed relatively quickly in both diluted and concentrated acid solutions, with samples collected after only 5 min already exhibiting appropriate positions of the 200 peak (7.8°). However, samples collected later than after 30 min did not contain any of the targeted phases, and it means ordered IWV-derived materials contain either −O– bridges or uniformly alternating −S4R–/–O– or −D4R–/–S4R– units in between crystalline layers. Instead of promoting a selective and uniform reconstruction of interlayer units, prolonging the treatment worsened layer crystallinity, as indicated by the broadened “intralayer” peaks and non-uniform transformation of S4Rs. In diluted acid solutions, further consumption of S4R interlayer units correlated with the shift of the 200 peak to the higher angles and with its broadening. In concentrated acid solutions, the 200 peak shifted to the lower angles in the patterns of treated samples, indicating partial, yet again, non-uniform, reconstruction of D4R units. Such a reconstruction was possible at the expense of atoms extracted from the framework at low pH or high temperature.^12,27^

**Figure 3:**
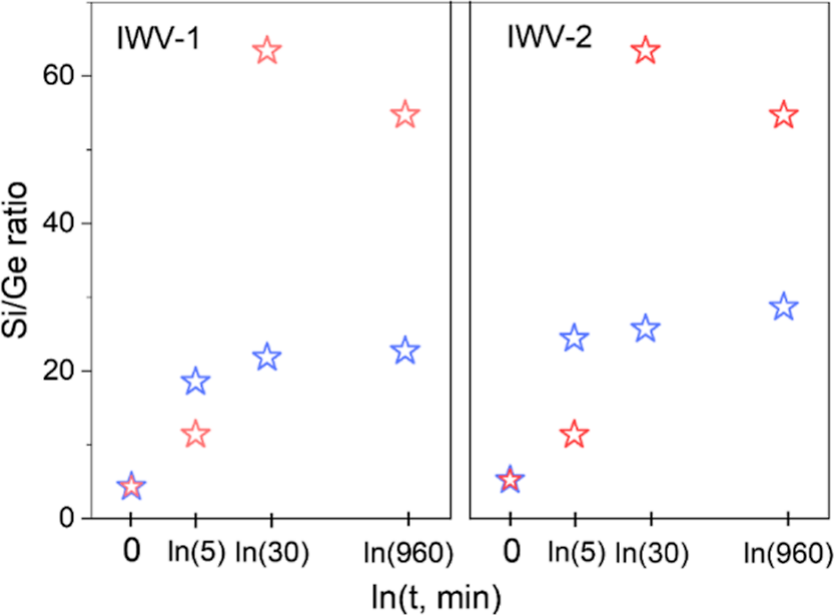
Chemical compositions of samples collected after different treatments. Si/Ge ratios of IWV-1 and IWV-2 treated with 0.1 M (blue) or 12 M HCl (red) for 5, 30, and 960 min.

The last two pathways to disordered materials, at the relatively high pH of a 0.1 M HCl (pH ≈ 1) solution or at the very low pH of a 12M HCl solution (pH ≈ −1), illustrate two oppositely directed processes in an acidic solution during ADOR, that is, the disassembly of germanosilicate units (D4R, S4R, and their combination) and the reconstruction of units with the same structure, but enriched with Si.^28^ Reconstruction requires increasing the mobility of Si atoms at pH < 0 (in 12 M HCl). At such a low pH, the initial disassembly (accompanied by a fast drop in Ge content, as shown in Figure 3) is compensated for by the reincorporation of framework atoms and thus the reconstruction of interlayer units (shift of the 200 peak to the left). The Ge content in the sample increases with the duration of the treatment due to the increased mobility of Ge species, most of which are dissolved in the solution (Figure 3). In contrast, the reconstruction of interlayer units is suppressed in a 0.1 M solution, furthering the disassembly (right shift of 200 peaks) and gradual depletion of the Ge fraction in the structure until plateauing (Figure 3). This evolution of Si/Ge upon different treatments is similar to that observed in samples with a different Ge content and therefore results from the treatment conditions, not from the chemical composition of the material.

Based on these findings, the transformation of IWV zeolite in diluted acid solutions was preferred to avoid uncontrollable reconstruction. However, two problems remained unsolved, namely, (i) the excessive rate of disassembly, which led to the consumption of the targeted intermediate phase (IPC-20), and (ii) the chemical lability of the layers. Nevertheless, specific stages of the multistep ADOR process can be decelerated by implementing specific conditions for transformation, as recently shown.^29^

**Figure 4:**
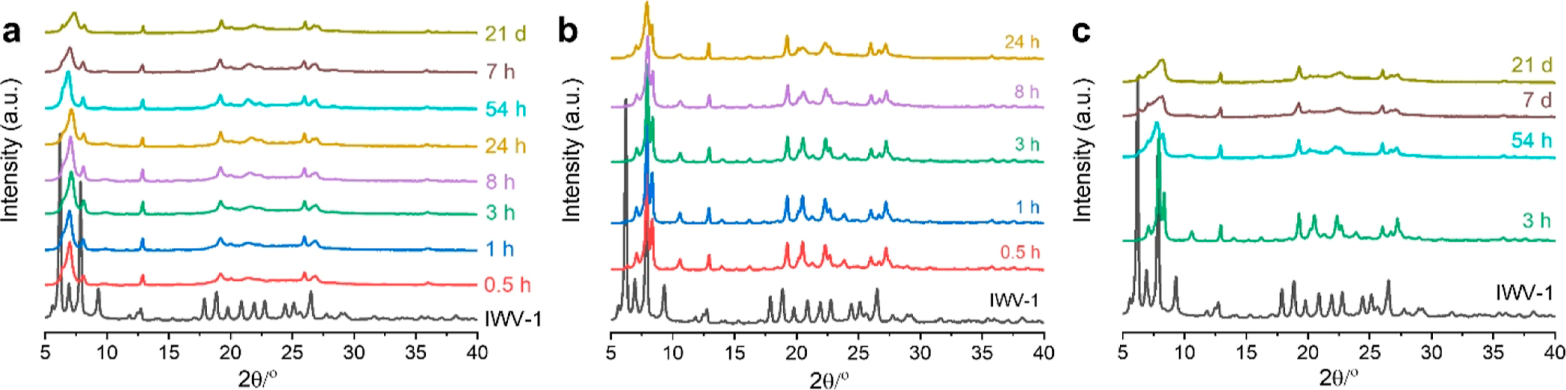
Evolution of the structural transformation of IWV-1 in a water–alcohol acid solution. (a) XRD patterns of the initial IWV-1 zeolite and disassembled samples prepared by treating IWV-1 with a 40 wt % MeOH/H_2_O solution at 60 °C for 0.5 h – 21 d. (b–c) XRD patterns of products after calcining the respective disassembled precursors at 450 °C for 2 h. All products prepared using a 40 wt % MeOH/H_2_O solution within 24 h had a new zeolite structure (IPC-20) (b), whereas prolonging the treatment led to a disordered structure (c).

Decreasing solvent polarity and the solubility of intermediate inorganic species by adding aliphatic alcohols enabled us to restrict both the extent of the structure disassembly and the susceptibility of the layers to hydrolysis (Figure 4a). Applying water–alcohol solutions to treat IWV under optimized conditions increased the lifetime of IPC-20 from 5–30 min up to 24 h (Figure 4b). Prolonging (>24 h) the treatment, conversely, led to disordered materials (samples collected at 54 h and later, Figure 4c) similar to those obtained in acid solutions (Figure 2). Ultimately, the Si/Ge ratio of IPC-20 prepared using the optimized procedure was virtually constant (around 12) between 0.5 and 8 h, indicating an almost complete suppression of disassembly or reconstruction of interlayer units. Ge atoms remained in IPC-20 formed by IWV hydrolysis in a water–methanol solution due to the slow reduction of D4R-to-S4R units, as postulated previously,^29^ rather than to the reincorporation of leached Ge, as observed in 12 M HCl. Taking into account the possibility to recover and recycle Ge leached upon IWV-to-IPC-20 transformation,^30^ the overall yield of solid IPC-20 product can be estimated as nearly quantitative (>95%).

### 2.3 Determination of the IPC-20 Structure

**Figure 5:**
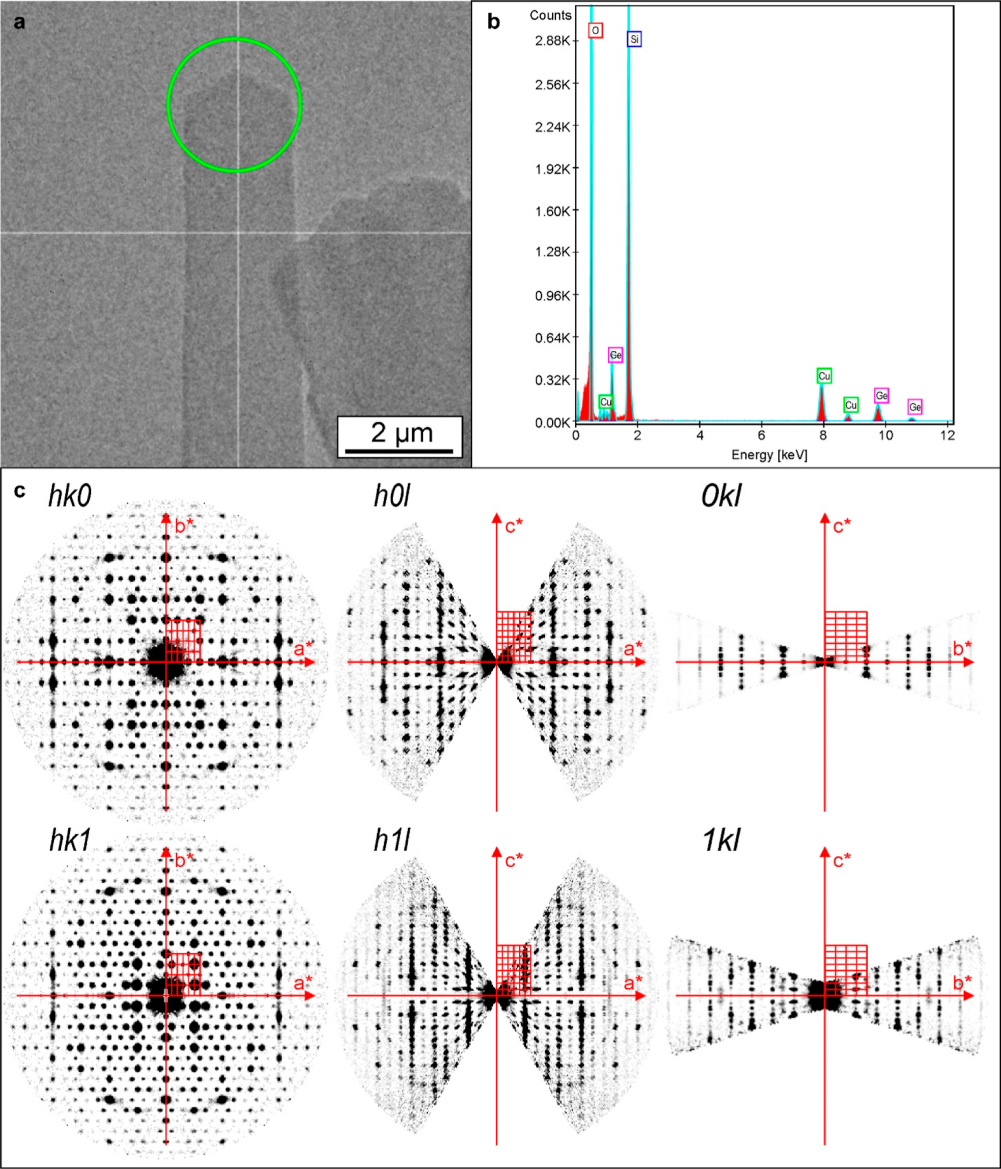
Structure determination by 3D ED. (a) Crystal selected for 3D ED data collection; the area measured is defined by the nano beam size (green circle). (b) EDS spectrum showing Ge in the crystal used for crystallographic analysis by 3D ED. (c) Sections of the reciprocal space reconstructed for the crystal.

To demonstrate the formation of zeolite IPC-20, presumably resembling the initial IWV with S4Rs instead of D4R interlayer units, we characterized its structure by precession-assisted three-dimensional electron diffraction (3D ED, see Figure 5)^31,32^ and X-ray powder diffraction (XRPD). As a method for structure determination from single micro- or nanocrystals (Figure 5a), 3D ED was chosen to characterize the structure of IPC-20 because this material did not form crystals large enough for X-ray single crystal diffraction, and its powder diagram had a low resolution.^33^ The resulting 3D ED data at 100 K indicated an orthorhombic unit cell with *a* = 25.356(4) Å, *b* = 13.812(2) Å, *c* = 22.718(6) Å, and *V* = 7954(3) Å^3^. The sections of the reciprocal space suggested *C*-centering (condition h + k = 2n on hkl), c-glide perpendicular to *b* (condition l = 2n on h0l), and e-glide (condition h,k = 2n on hk0) (Figure 5c). These 3D ED data matched the space groups *Cmce* and *C*2*ce*, in line with the extinctions observed in XRPD.

The structure of IPC-20 was solved using direct methods with SIR2014 from data with 75% completeness up to the resolution of sinθ/λ = 0.55 Å^–1^.^34^ Despite combining several data sets, as usual with 3D ED data, we were unable to compensate for the limited data completeness due to the strong (001) preferential orientation of the crystals on the grid. The complete structure could only be solved in the non-centrosymmetric space group *C2ce*. The structure model contained 52 independent atoms, namely, 10 silicon sites and 42 oxygen sites. The refinement was performed against 3D ED data considering both the kinematical approximation (fast) and dynamical effects (more accurate but computationally time-consuming). Only Si was considered in the refinements in Si/Ge sites as the structure solution did not show any preferential position for Ge.

The kinematical refinement converged to *R/*wR(obs) = 0.2244/0.2873 and *R/*wR(all) = 0.2856/0.3020 for 2446/4310 observed/all reflections and 183 refined parameters. The dynamical refinement led to *R/*wR(obs) = 0.1159/0.1202 and *R/*wR(all) = 0.2793/0.1423 for 3894/19857 observed/all reflections and 273 refined parameters. Given the limited data coverage and rather low resolution, Si–O and O–O distance restraints were applied to maintain the tetrahedral geometry of the SiO_4_ units in both refinements. The most important 3D ED experimental and refinement parameters are listed in Table S1. The new zeolite structure IPC-20 derived by 3D ED was saved as a CIF file and deposited in the Cambridge Crystallographic Data Centre (CCDC) database under deposition number 2203343. The structural parameters are summarized in Table S2.

**Figure 6:**
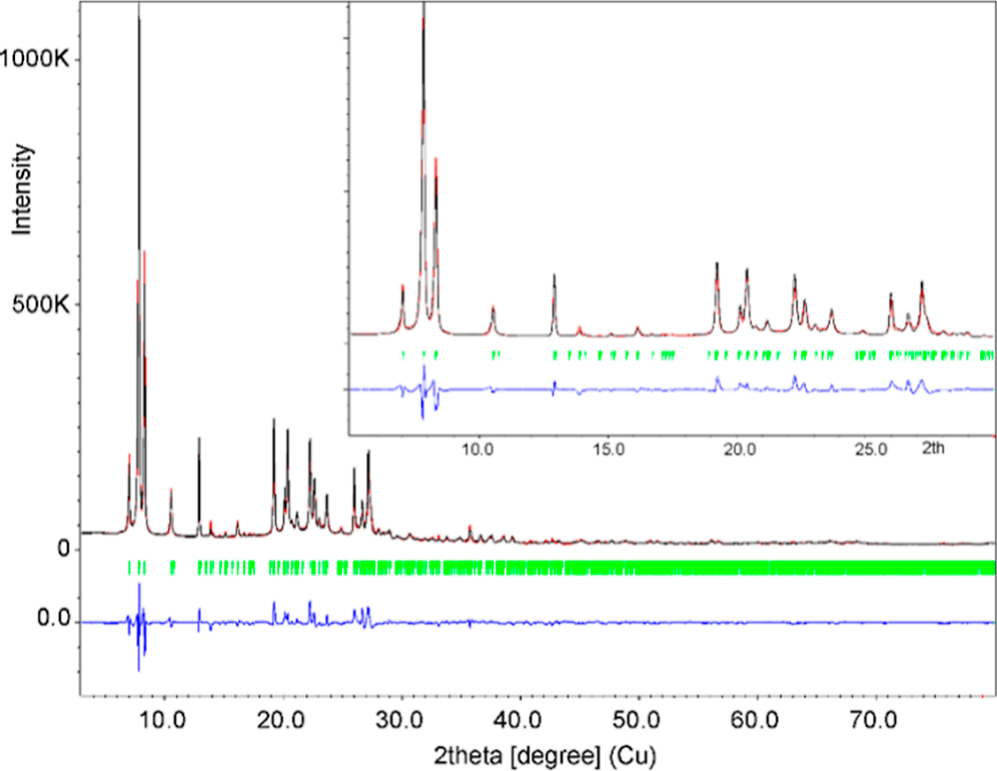
Rietveld refinement from X-ray powder diffraction at room temperature. The diagram shows calculated (red), measured (black), and difference (blue) curves. The reflection positions (Kα1/Kα2) are indicated by the red ticks below the graph.

The structure obtained at the nanoscale at −173 °C was confirmed at a larger scale by Rietveld refinement from XRPD data collected at room temperature (Figure 6): *R/*wR(obs) = 0.0309/0.0370 and *R/*wR(all) = 0.0309/0.0370, *R*p = 0.0611, w*R*p = 0.0829, and GoF = 0.1426 for 1908/1908 observed/all reflections and 161 refined parameters (detailed parameters in Table S3). The residual solvent in the pores at room temperature could not be properly described to explain the values of the profile parameters. The Rietveld refinement provided accurate lattice parameters for IPC-20 at room temperature: *a* = 25.0982(8) Å, *b* = 13.7003(3) Å, *c* = 22.5228(7) Å, and *V* = 7744.6(4) Å^3^.

**Figure 7:**
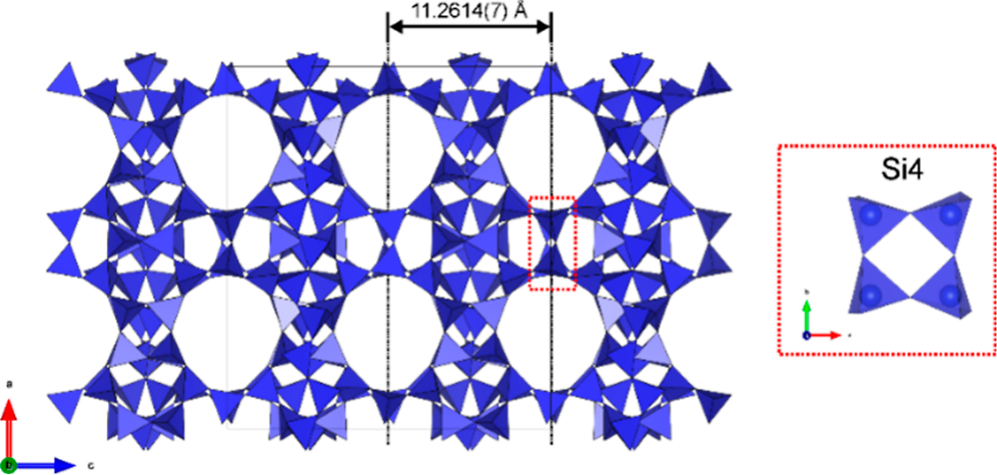
IPC-20 structure solved by dynamical refinement of 3D ED data. The S4R unit that forms the bridge between denser layers is shown in the red inset.

The IPC-20 structure presented the expected S4R units instead of D4R units of the parent material (Figure 7). The denser silicate layers with a topology similar to those of IWV layers were connected along the *c*-axis to more disordered S4R units via their vertices, forming a one-dimensional, straight channel down the *b*-axis. The S4R units were disordered, as shown by the significantly higher atomic displacement parameters of the atoms of these units. The interspacing between two consecutive layers in IPC-20 was *c*/2 = 11.2614(7) Å at room temperature, confirming structural shrinkage along the *c*-axis in relation to the parent IWV. This crystallographic study demonstrated that the ADOR transformation replaced D4R units by S4R units while preserving the topology of the main silicate layers.

EDS analysis performed on the same crystal used for 3D ED identified Ge with an approximate Si/Ge ratio of 12.3 (Figure 5b). However, the low resolution of the 3D ED data (sinθ/λ(max) = 0.55 Å^–1^), together with the limited data coverage (75%) and partial disorder, hampered the accurate localization and refinement of the Si/Ge ratio in S4R units. The kinematical and dynamical refinements unexpectedly showed that the Si sites of the S4R units (Si4_1 and Si4_2) likely contained Ge, with the highest atomic displacement parameters. These parameters reflected the higher disorder of S4R units dangling between two denser silicate layers and therefore hiding the Ge atoms. Such a disorder increases with the duration of the treatment (Figure 2). The presence of Ge was also evidenced by the higher Si–O distances on sites of S4R units substituted by Ge. However, the low data coverage and slightly disordered S4Rs prevented us from precisely assessing Si–O distances to determine the Ge localization.

**Figure 8:**
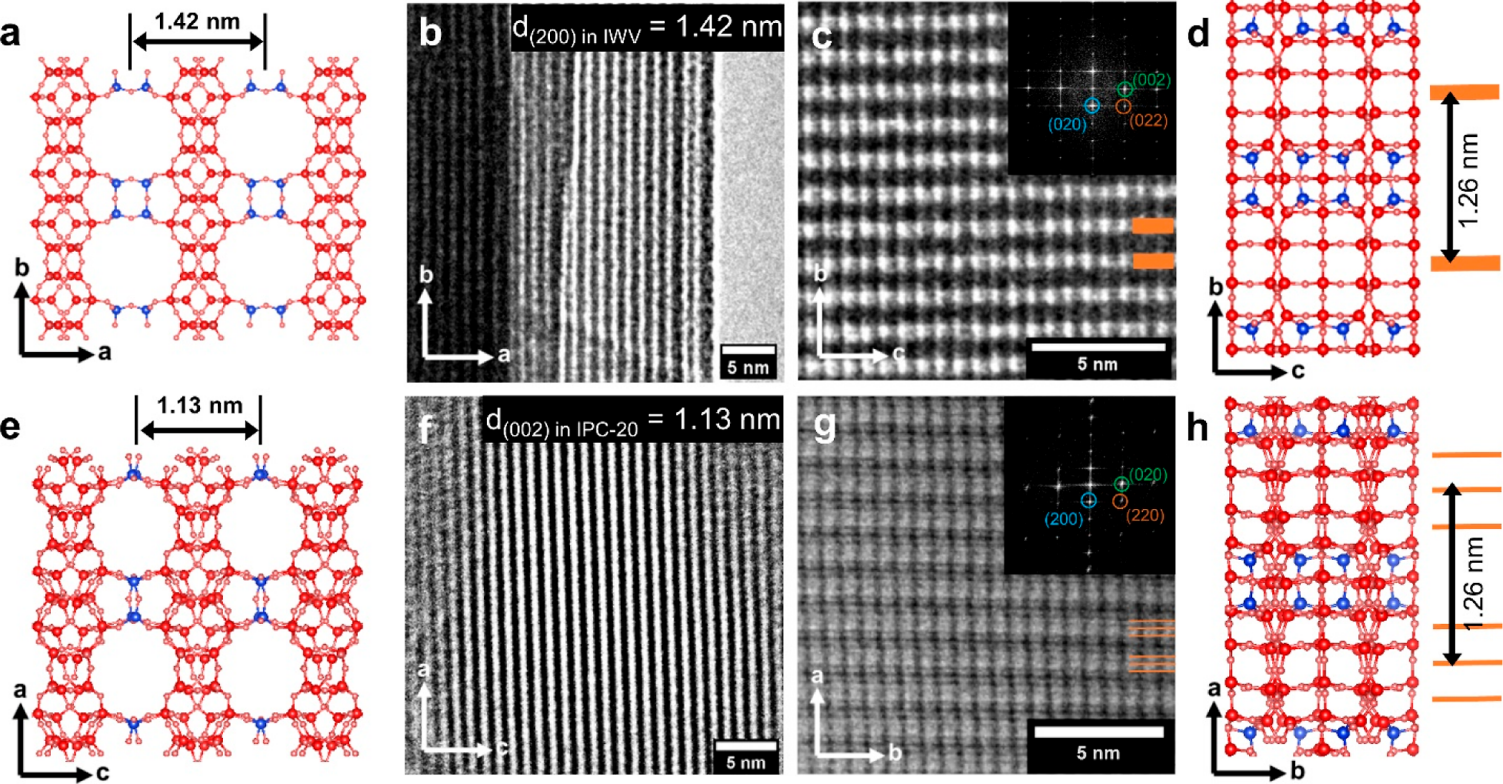
Crystallographic models and corresponding TEM images of parent IWV and its daughter zeolite IPC-20. The *ab* projection of IWV and the *ac* projection of IPC-20 (“side” view) show the layer connectivity (a–b for IWV, e–f for IPC-20), whereas the *bc* projection of IWV and the *ab* projection of IPC-20 (“top” view) identify the layer plane that remains unchanged during the ADOR structural transformation (c–d for IWV and g–h for IPC-20). The D4R units in IWV zeolite convert into S4R units, thereby decreasing the interlayer distance from 1.42 to 1.13 nm. In the models, T atoms in D4R and S4R units are highlighted in blue.

The structural transformation of IWV into IPC-20, which affected the lattice parameters in some directions, was also confirmed by the high-resolution transmission electron microscopy (HRTEM) results (Figure 8): (i) decrease in *d*_200_-spacing from 1.42 to 1.13 nm (Figure 8 a–b,e–f) and (ii) similar topology of layers with *d*_020_-spacings around 1.26 nm, in line with the respective IWV (Figure 8c–d) and IPC-20 (Figure 8g–h) models. In addition, preservation of the layers was confirmed by applying fast Fourier transform (FFT) to images of the layers, clearly showing that spots corresponding to the 002, 020, and (022) planes matched. Accordingly, notwithstanding the structural changes resulting from the IWV-to-IPC-20 transformation, the crystal morphology remained virtually the same (Figures S8 and S9).

Changes in the IWV structure were also reflected in the pore system of IPC-20. The straight 12-ring channels (pore size: 6.9 × 6.2 Å) connected by 12-ring openings of the parent IWV^35^ become 10-ring channels as interlayer connecting units contract in the IWV-to-IPC-20 transformation. This contraction decreases the micropore volume and average micropore size without generation of other types of pores (such as mesopores, defects, and interparticle voids, to name a few), which often occurs under acidic treatment.^36^

**Figure 9:**
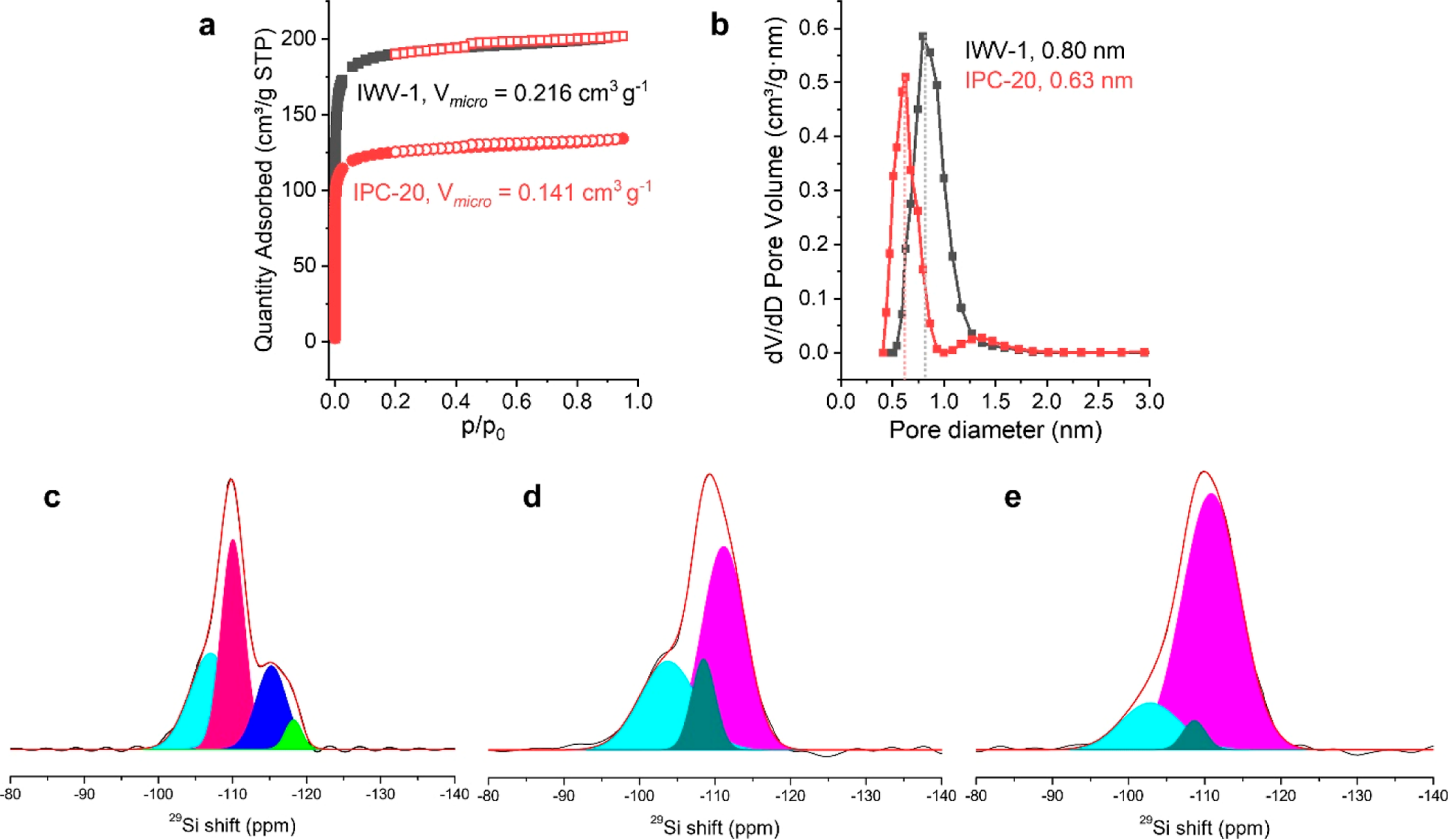
Characterization of micropores and Si atom environments in IWV-1, precursor, and IPC-20. Ar adsorption/desorption isotherms (a) and pore size distribution (b) of parent IWV-1 and daughter IPC-20 zeolites and ^29^Si MAS NMR spectra of IWV-1 (c), IPC-20 precursor (d), and final IPC-20 zeolites after condensation at 450 °C for 2 h (e).

The micropore volume of IPC-20 was lower than that of the starting zeolite, 0.141 vs 0.216 cm^3^ g^–1^, respectively. In turn, the shape of the IPC-20 and IWV adsorption isotherms did not significantly differ (Figure 9a), indicating that no new pores with sizes >2 nm were formed in the transformation. The presence of pores of various sizes and their interconnectivity in both parent and daughter zeolites makes it difficult to differentiate individual pores in these materials. However, the average pore size, which reflects changes in the size of individual channels, can still be estimated from pore-size distribution plots (Figure 9b). The average channel diameter decreased from 0.80 nm in IWV to 0.63 nm in IPC-20.

^29^Si MAS NMR spectroscopy showed changes in the environment of Si atoms associated with alterations in the structural fragments linked to these atoms. The parent IWV-1 (Figure 9c) exhibited signals related to three types of Si atoms (at −107 ppm, −110 ppm, and in the region from −115 to −118 ppm). Peaks at −107 and −110 ppm were associated with Q^4^ silicon atoms surrounded by different amounts of Ge atoms, i.e., corresponding to the species Si(OSi)_*n*_(OGe) _(4–*n*)_ with variable *n*,^19^ whereas signals in the region from −115 to −118 ppm were attributed to Si atoms in D4R units.^37^

Upon structural transformation of the IWV zeolite, which resulted in the destruction of D4R units and removal of Ge atoms, the intensity of the peaks corresponding to Si–O–Ge connections (either in layers or interlayer units) significantly decreased (Figure 9d). Signals in the region −115–(−118) ppm disappeared, suggesting the complete removal (rearrangement) of the initial interlayer connections. Instead, a new peak appeared at −103 ppm, corresponding to the large fraction of silanol defects formed during the hydrolysis of Ge–OSi bonds in IWV. In the subsequent condensation of silanols (Si–OH + HO–Si → Si–O–Si), deficient Si(OSi)_3_OH groups were transformed into Si(OSi)_4_ species, with a decrease in the intensity of Q^3^ signals (−103 ppm) and an increase in the intensity of Q^4^ peaks (−111 ppm, Figure 9e). However, not all Q^3^ signals disappeared once the IPC-20 zeolite was formed, suggesting that a fraction of silanol defects formed during hydrolysis was maintained in the final sample. In general, the evolution of the signals in ^29^Si NMR spectra supported the proposed mechanism of IWV transformation into the novel IPC-20 material.

## 3 Conclusions

IWV belongs to a family of zeolites with an untapped potential for transformation by ADOR. As with many other chemically labile framework materials, including germanosilicates, IWV is susceptible to water. The output of IWV water-assisted deconstruction depends on the intrinsic characteristics of the parent zeolite, such as the Si/Ge ratio in the framework and the morphology of its crystals. Furthermore, this output can be controlled by balancing the reaction rates of sub-processes (D4R unit dissolution and layer degradation and reconstruction) upon structural conversion. A phase of the new zeolite with a framework structurally related to the initial IWV can be “caught” by optimizing the design of the starting zeolite and by varying synthesis parameters. Using seed-assisted crystallization of IWV followed by its gentle transformation in the water–alcohol system, the highly crystalline daughter zeolite IPC-20 is produced, as shown by solving its structure using 3D ED. The IPC-20 structure contains S4R units instead of the D4R units of the parent material, but the dense silicate layers remain virtually intact. Adsorption and spectroscopic characterization demonstrate negligible layer destruction phenomena upon the whole sequence of transformations. These findings demonstrate the efficiency of this post-synthesis method for preparing highly crystalline zeolites based on the transformation of chemically labile frameworks.

## 4 Experimental Section

### 4.1 Synthesis of 1,5-Bis(tetramethylimidazolium)pentane Bromide

The 1,5-bis(tetramethylimidazolium)pentane (BTP) cation was used as the organic structure directing agent (OSDA) to synthesize IWV zeolites.^18,19^ Typically, 25 g of 1,2,4,5-tetramethylimidazole (98%, TCI Chemicals) and 21 g of dibromopentane (98%, TCI Chemicals) were mixed in 100 ml of methanol and then refluxed for 4 days. When the reaction was finished, the gelatinous product in the bromide form (BTPBr) was formed at the bottom of the flask, which was separated by pouring out the upper methanol. The product was washed with methanol and diethyl ether and then dried in a vacuum oven at room temperature. The OH form of BTP (BTPOH) was obtained by ion exchange with Ambersep 900(OH) (exchange rate 67.4%), which was used as the OSDA to subsequently prepare IWV zeolites.

### 4.2 Synthesis of IWV Germanosilicate Zeolites

IWV-3 (3 represents the Si/Ge ratio in the synthesis gel) zeolite was prepared with a gel composition of 1.0 SiO_2_/0.33 GeO_2_/0.25 OSDA/20–30 H_2_O based on a modified protocol.^19^ Typically, 0.69 g of GeO_2_ (99.99%, Sigma-Aldrich) was dissolved in 26.73 g of BTPOH solution (exchange rate 67.4%), subsequently adding 4.17 g of TEOS tetraethyl orthosilicate (TEOS, Sigma-Aldrich) and stirring at room temperature to hydrolyze TEOS and evaporate water to the desired gel composition. The gel was transferred to a Teflon container, charged into an autoclave, and then hydrothermally treated statically at 175 °C for 14 days. After the reaction, the autoclave was cooled down under tap water. The product was washed out with distilled water and ethanol by filtration. The occluded OSDA was removed by calcination at 550 °C for 6 h. IWV-3 was further used as seeds to synthesize IWV-1 and IWV-2. The gel compositions of IWV-1 and IWV-2 were similar to those of IWV-3, albeit with different Si/Ge ratios and additional seeds of IWV-3 (2 wt % seeds/TO_2_). The crystallization time of IWV-1 and IWV-2 was shortened to 10 days. The remaining preparation and OSDA removal procedures were the same as those described for IWV-3.

### 4.3 Post-treatment of IWV Zeolites

In total, 0.1 g of IWV zeolite was added into 10 mL of HCl solution (0.1, 6 M, or 12 M) and stirred at room temperature or 100 °C for specific times. Then, the disassembled precursor was separated by filtration, washed out with anhydrous ethanol, and dried at 60 °C overnight. Finally, the precursor was calcined at 450 °C for 2 h with 1 °C min^–1^. In turn, 0.6 g of IWV-1 zeolite was added into 96 mL of a MeOH/H_2_O (40 wt %) solution and stirred at 60 °C. When reaching the desired time, a 10–15 mL solution was elicited, filtrated, and washed with anhydrous ethanol. After drying at 60 °C overnight, the precursor was calcined at 450 °C for 2 h with 1 °C min^–1^.

### 4.4 Characterizations

The crystallinity of all samples was determined by XRD on a Bruker D8 Advance diffractometer with a graphite monochromator and Cu_Kα_ radiation in Bragg–Brentano geometry. The morphologies were determined under an FEI Quanta 200F scanning electron microscope equipped with a wavelength-dispersive X-ray detector (WDS). Samples were placed on the conducting carbon tape without any metal coating prior to the measurement. Besides, a JEOL JSM-IT800 Schottky field emission scanning electron microscope was also employed for morphology examination. Ar adsorption/desorption isotherms were collected at −186 °C on a 3Flex (Micromeritics) static volumetric apparatus. All samples were degassed on a SmartVac Prep (Micromeretics) at 300 °C under vacuum for 8 h before the sorption measurements. The surface area was calculated using the BET method and adsorption data in a relative pressure range of *p*/*p*0 = 0.05–0.25. The *t*-plot method was applied to determine the micropore volume (*V*_micro_). The pore size distributions were calculated using the density functional theory (DFT) method.

The chemical composition of the studied zeolites was determined by ICP–MS analysis (Agilent 7900 ICP–MS system), otherwise EDS. 50 mg of the sample was mixed with 1.8 mL of HNO_3_ (67–69%, ANALPURE), 5.4 mL of HCl (34–37%, ANALPURE), and 1.8 mL of HF (47–51%, ANALPURE), then transferred into a closed Teflon vessel (60 ml, type DAP60), placed in the microwave (Speedwave XPERT, Berghof), and heated at 210 °C (5 °C min^–1^) for 25 min. After cooling down, the complexation of the surplus HF was done by adding 12 mL of H_3_BO_3_ and further treatment in the microwave at 190 °C (5 °C min^–1^) for 10 min. Finally, the obtained cooled down solutions were diluted for analysis.

Magic angle spinning (MAS) ^29^Si and ^19^F NMR spectra were recorded on a Bruker Advance III HD spectrometer in a magnetic field of 11.75 T corresponding to a ^29^Si Larmor frequency of 99.4 and 470.9 MHz for ^29^Si and ^19^F, respectively, at room temperature. The samples were packed into 2.5 mm ZrO_2_ MAS rotors rotating at 20 kHz MAS speed. Considering the small amount of material that fits in the rotor, as well as the low natural abundance of ^29^Si, a large number of signal scans were required for an acceptable signal-to-noise ratio of the spectra. This limitation, combined with the long relaxation delay between scans, rendered a regular single-pulse acquisition of spectra impracticable in terms of experiment time. Therefore, a multiple-echo acquisition was employed, with a pulse sequence , which enabled us to accumulate up to *n* = 256 whole echoes within a scan. The ^19^F signals (free induction decays) were acquired following a single 90°-pulse, with 1280 signal repetitions at 30 s intervals.

The acquisition window *acq* was set to 2.5 ms to accommodate the whole echo without much of a signal truncation. The number of scans was 720, and the interval between scans was 1 min; hence, the total experiment time was 12 h per sample. The acquired echoes were summed up and processed into a real spectrum using the NMR data-processing program ssNake.^38^ The actual number of echoes included in the sum varied with the effect of *T*_2_ relaxation on the ^29^Si NMR lineshape in each sample. The spectrum of IWV-1 retained its shape during *T*_2_ relaxation, thus enabling us to include all available echoes. In IPC-20P and IPC-20, conversely, *T*_2_ relaxation introduced a noticeable bias in the lineshape; therefore, to preserve the quantitative character of the lineshape analysis in those samples, the summation was limited to the first echoes. The ^29^Si NMR spectra were decomposed into up to four Gaussian components, varying all parameters of the Gaussian function—center frequency, width, and area. During the decomposition, the width parameters were restricted to 10 ppm.

Transmission electron microscopy (TEM) images were acquired using a JEOL NEOARM 200 F microscope equipped with a Schottky-type field emission gun at an accelerating voltage of 200 kV and TVIPS TemCam-XF416 CMOS camera. Scanning images (STEM) were collected with annular dark-field (ADF) or annular bright-field (ABF) detectors. Samples were finely grinded and deposited onto holey carbon films supported on copper grids.

3D ED data were collected on an FEI Tecnai G2 20 transmission electron microscope (acceleration voltage of 200 kV, LaB_6_) equipped with a side-mounted hybrid pixel detector ASI Cheetah M3, 512 × 512 pixels with high sensitivity and fast readout. The powder sample was sprinkled on a Cu grid coated with a thin film of holey amorphous carbon. To preserve the possible pore content under high vacuum in TEM, the grid was plunged into liquid nitrogen and transferred to the transmission electron microscope using a Gatan cryo-transfer holder. To further mitigate the dynamical effect, 3D ED^31,32^ was coupled with precession electron diffraction (PED) using the precession device Nanomegas Digistar.^39^ The precession semiangle was set to 1°. For each selected crystal area (Figure 5a), non-oriented patterns were sequentially collected in steps of 1° in the accessible tilt range of the goniometer using the in-house software RATS while tracking the crystal following the procedure described by Plana-Ruiz et al. (2020).^40^ Low illumination settings were used to limit beam-induced damage to crystals. Data reduction was performed using the computer program PETS2.^41^ Most data sets presented a resolution below sinθ/λ = 0.5 Å^–1^, and the data set with the best resolution (sinθ/λ(max) = 0.55 Å^–1^) was used in the structural analysis. The result of the data reduction was an hkl-type file (*R*_int_(obs/all) = 0.1551/0.1783), which was used in the structure solution and kinematical refinement. For dynamical refinement, another hkl-type file was produced in which each ED frame was considered independent.^42^ The structure was solved using SIR2014^34^ in Jana2020^43^ and refined using DYNGO and Jana2020. The data collection details are presented in Table S1. The TEM was equipped with an energy-dispersive analyzer EDAX Octane silicon drift detector (SDD). By EDS analysis, Si, O, and Ge at a Si/Ge = 12.3 were detected in the ICP-20 crystal where 3D ED data were previously collected. The Cu signal present in the spectrum (Figure 5b) stems from the sample support.

The sample was ground and placed in the 0.5 mm borosilicate-glass capillary. Powder diffraction data were collected using the Debye–Scherrer transmission configuration on the powder diffractometer Empyrean of PANalytical (λ_Cu,Kα_ = 1.54184 Å) equipped with a focusing mirror, capillary holder, and PIXcel3D detector. The 20 h-long measurement was made from 4 to 80° 2Theta with a 0.013° step size and 3000 s/step. These powder data were used in the Rietveld refinement.

## Supporting Information Available

XRD patterns, SEM images, NMR spectra, Ar adsorption/desorption isotherms, and structure parameters (PDF)
