# Supplementary material for: Catching a New Zeolite as a Transition Material during Deconstruction
Source: J Am Chem Soc. 2023 Apr 11;145(16):9081–91. doi: 10.1021/jacs.3c00423 (PMC10141410; doi:10.1021/jacs.3c00423)
Supplement: Supplementary file 1 — ja3c00423_si_001.pdf [file ja3c00423_si_001.pdf]

Supplemental information

Catching a new zeolite as a transition material during deconstruction

Qiudi Yue[1], Gwladys Steciuk[2], Michal Mazur[1], Jin Zhang[1], Oleg Petrov[3], Mariya Shamzhy[1], Mingxiu Liu[1], Lukáš Palatinus[2], Jiří Čejka[1], and Maksym Opanasenko[1]*

[1] Department of Physical and Macromolecular Chemistry, Faculty of Science, Charles University, Hlavova 8, 128 43 Prague 2, Czech Republic

[2] Institute of Physics, Academy of Sciences of the Czech Republic, v.v.i., Na Slovance 2, 182 21 Prague 8, Czech Republic

[3] Department of Low-Temperature Physics, Faculty of Mathematics and Physics, Charles University, V Holešovičkách 2, 180 00 Prague 8, Czech Republic

Correspondence: maksym.opanasenko@natur.cuni.cz

Supplementary Figs

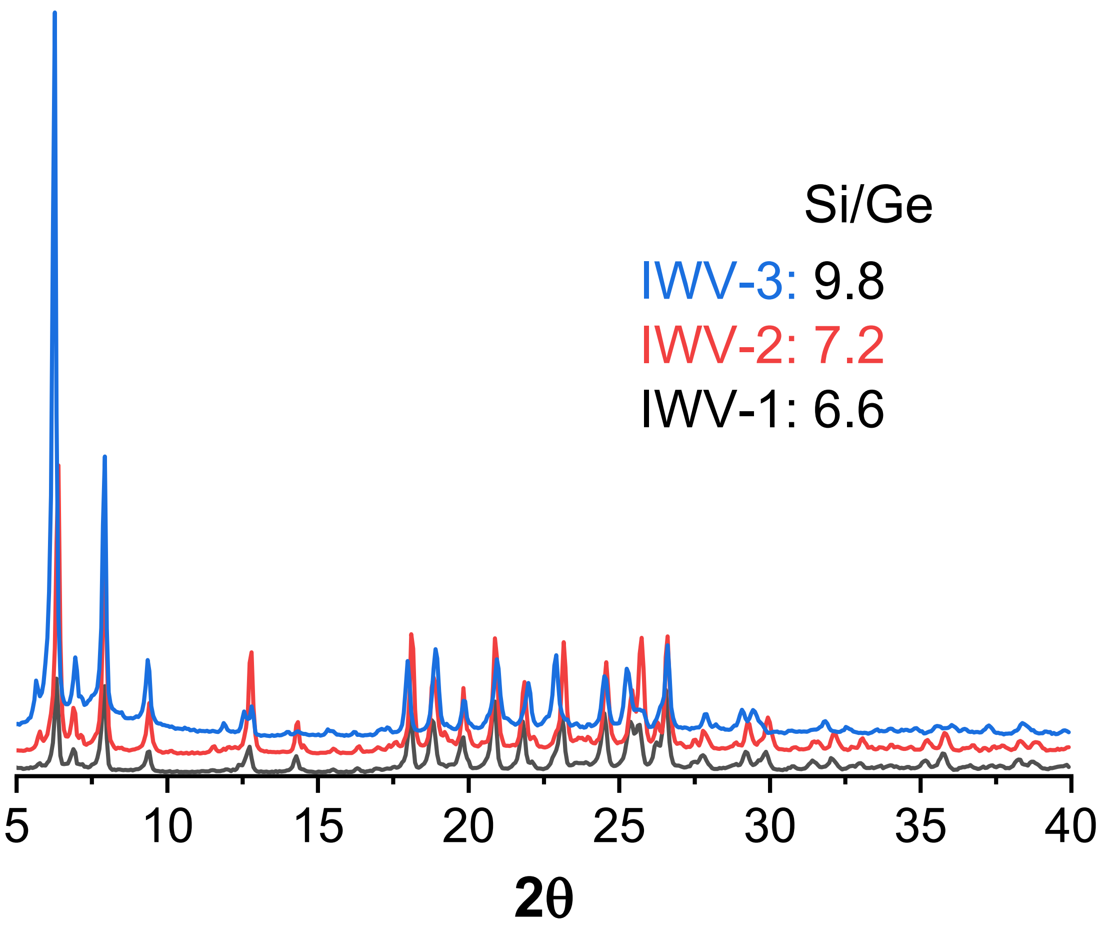

Figure S1. XRD patterns of IWV germanosilicate zeolites synthesized in the reaction mixture of Si/Ge =1-3 without seeding. The insert Si/Ge ratios of respective samples are determined by EDS.

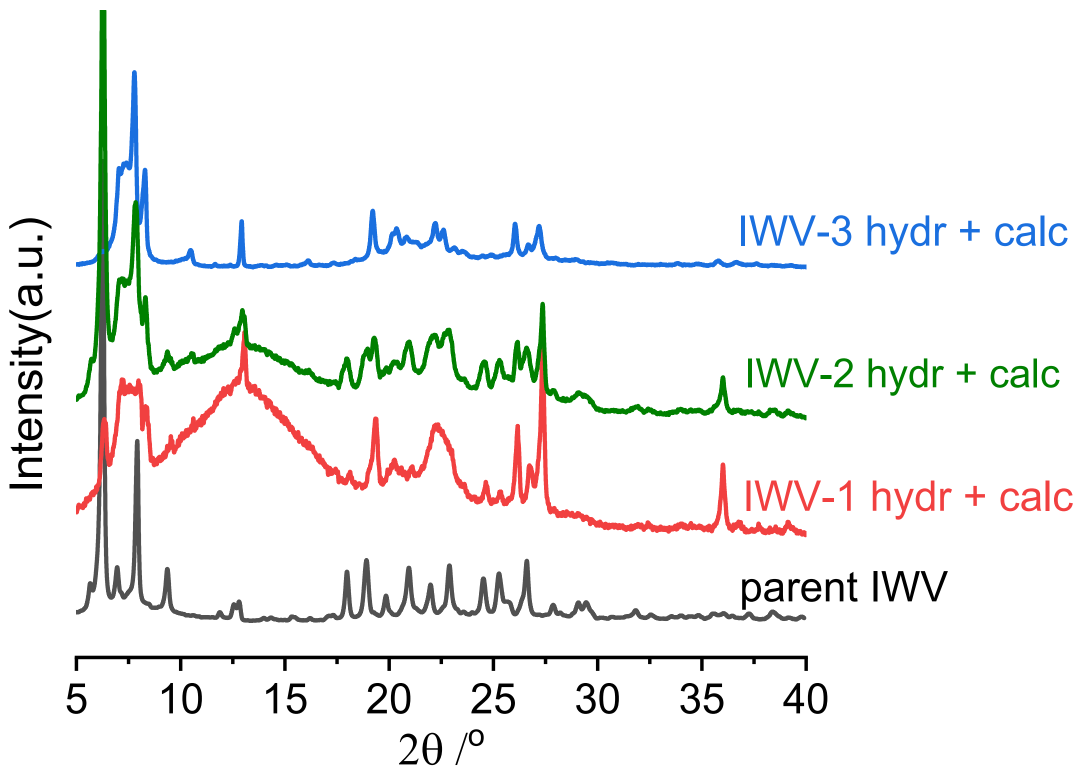

Figure S2. XRD for material obtained without seeds

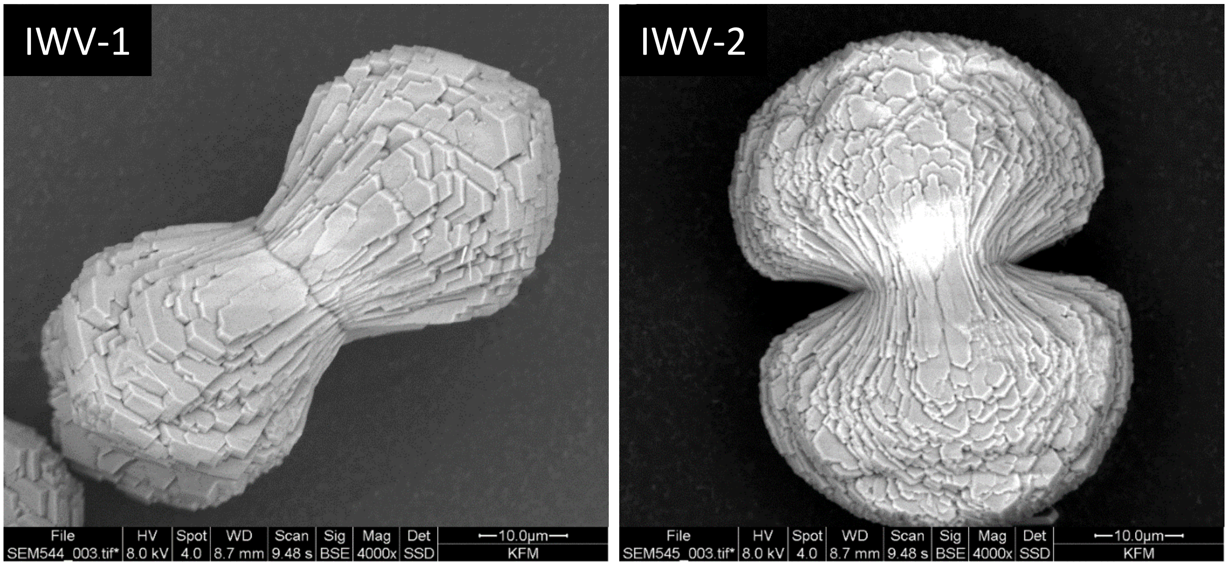

Figure S3. SEM images of IWV-1 and IWV-2 synthesized without seeding.

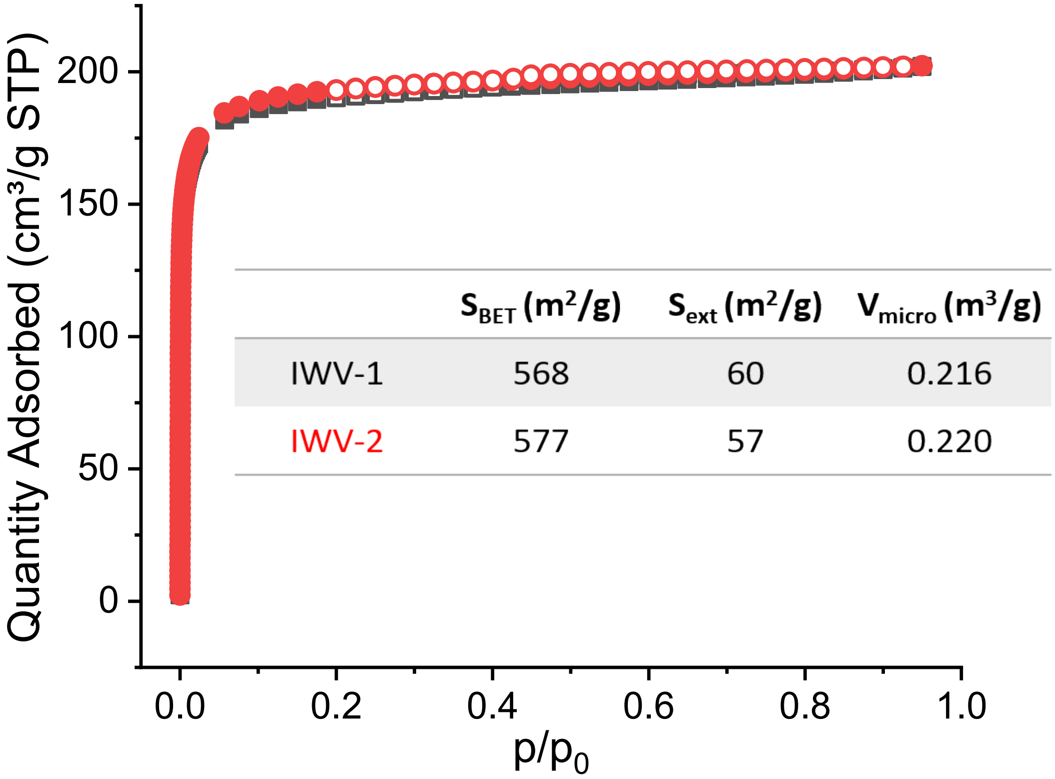

	S_{BET} (m^2/g)	S_{ext} (m^2/g)	V_{micro} (m^3/g)
IWV-1	568	60	0.216
IWV-2	577	57	0.220

Figure S4. Ar adsorption/desorption isotherms of IWV-1 and IWV-2 synthesized with seeding.

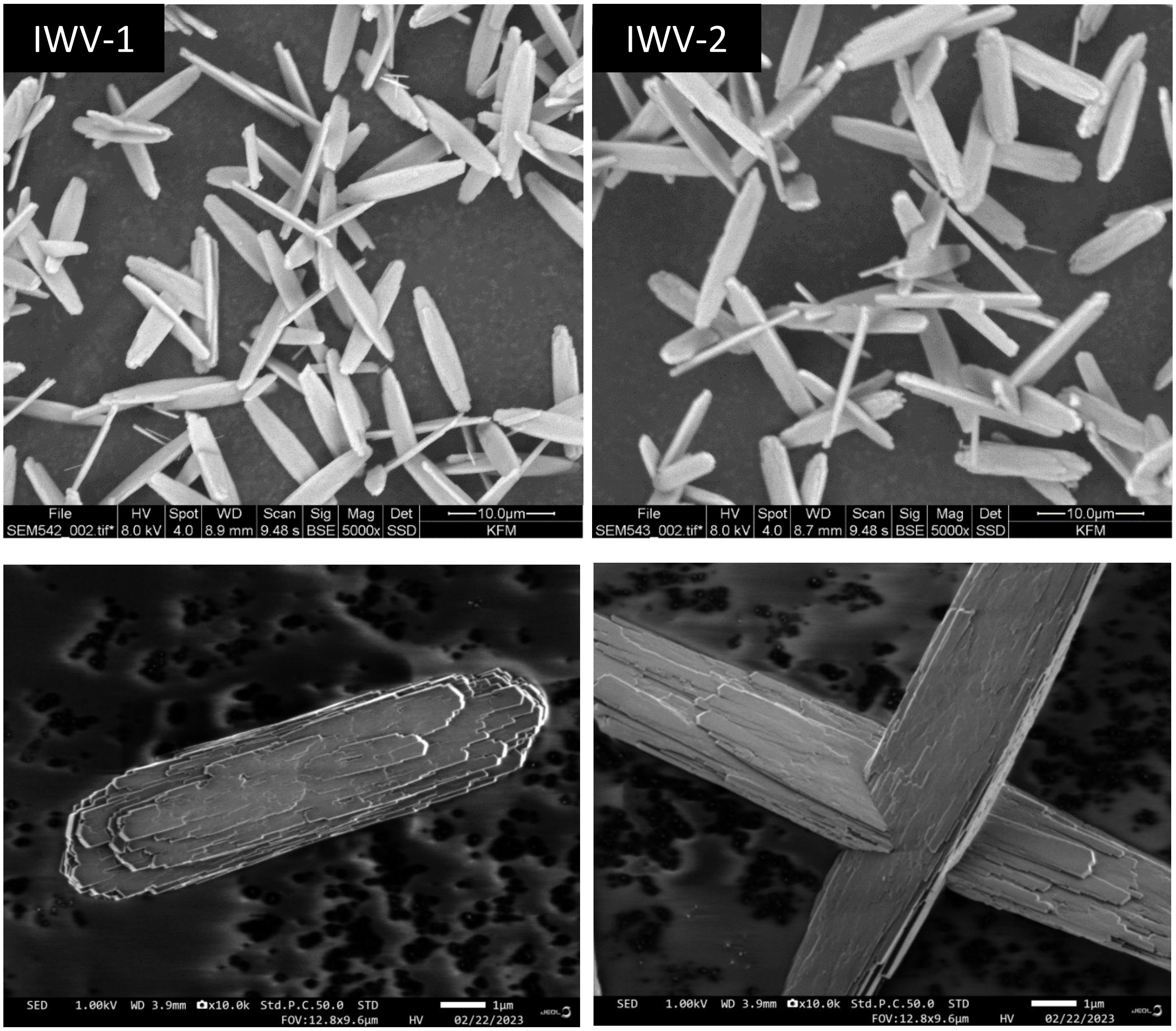

Figure S5. SEM images of IWV-1 and IWV-2 synthesized with seeding (top). Higher magnification SEM images of IWV-1 showing isolated and intergrown particles (bottom)

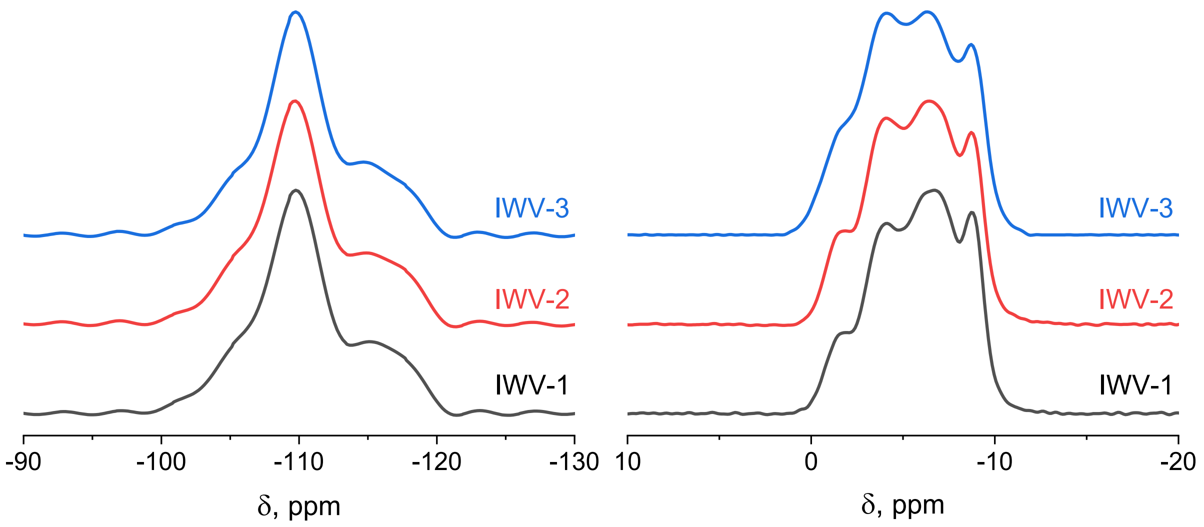

Figure S6. ^{29}Si (left) and ^{19}F (right) MAS CPMG NMR spectra of IWV-1, IWV-2 and IWV-3. The samples for ^{19}F NMR measurements were prepared by calcining the mixtures of NH_4F and OSDA-free zeolites at 60 °C for 12h, according to the reported reference (Angew. Chem., Int. Ed. 2011, 50, 5900).

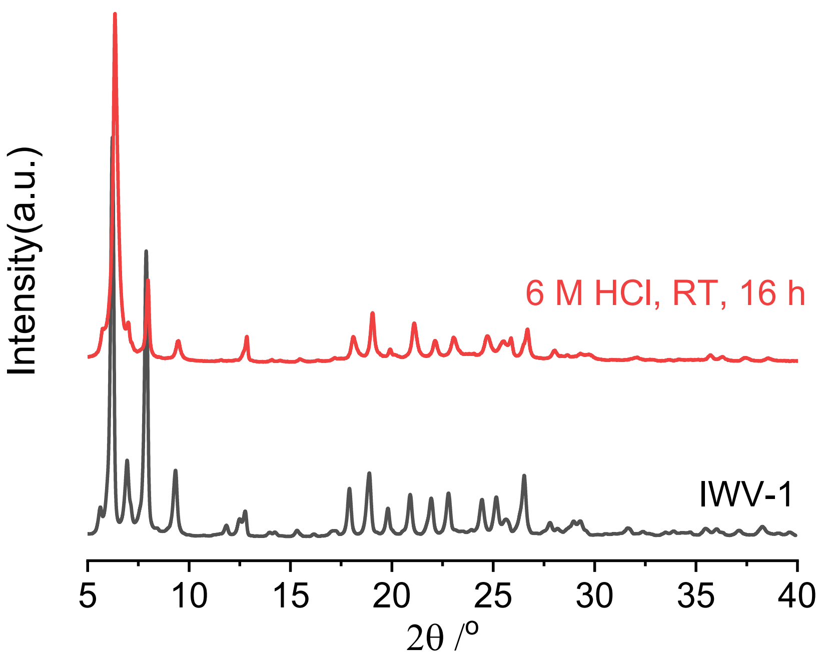

Figure S7. XRD patterns of IWV-1 and the product of its hydrolysis in 6 M HCl at RT for 16 h

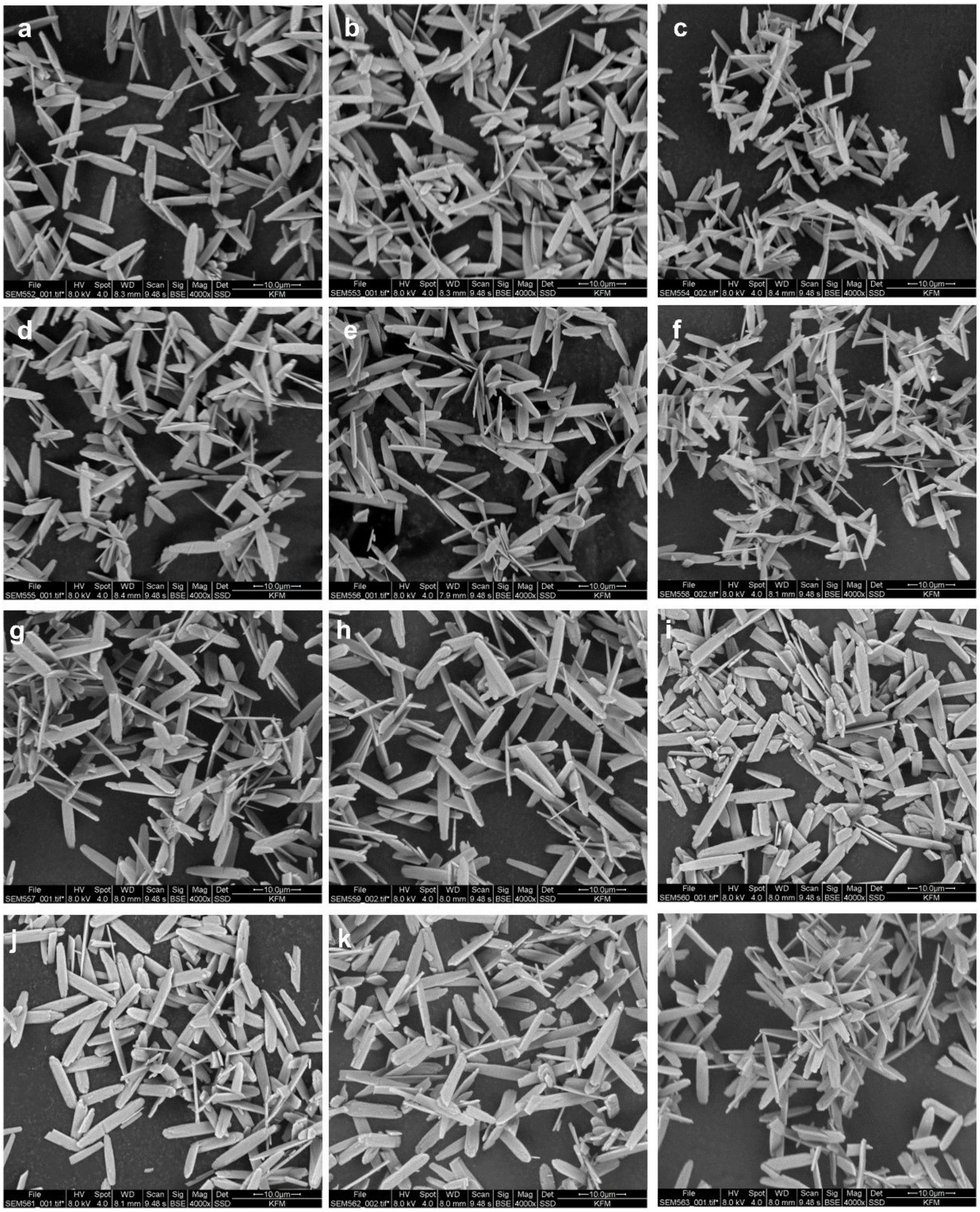

Figure S8. SEM images of

IWV-1 treated in 0.1 M HCl for 5 min (a), 30 min (b) and 16 h (c);

IWV-1 treated in 12 M HCl for 5 min (d), 30 min (e) and 16 h (f);

IWV-2 treated in 0.1 M HCl for 5 min (g), 30 min (h) and 16 h (i);

IWV-2 treated in 12 M HCl for 5 min (j), 30 min (k) and 16 h (l).

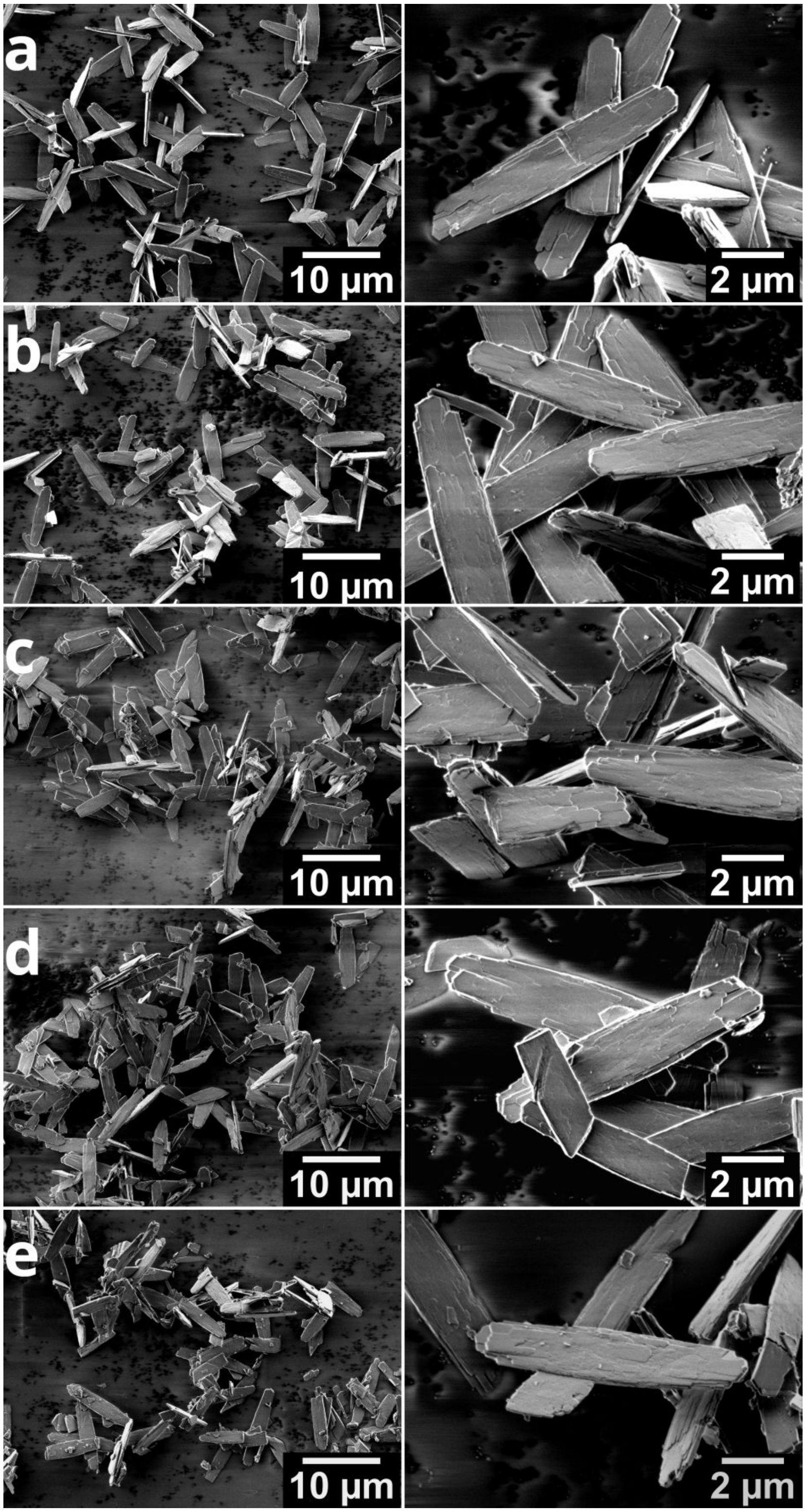

Figure S9. SEM images of IWV-1 treated in 40 wt. % MeOH/H_2O solutions for 30 min (a), 24 h min (b), 54 h (c), 21 d (d), and 26 d (e).

Supplementary Tables

Table S1. 3D ED data collection and structure refinement details at 100K

Refined structural formula	$(SiO_2)_{34}$
Crystal system	orthorhombic
Space group	*C*2*ce*
a	25.356(4) Å
b	13.812(2) Å
c	22.718(6) Å
V	7954(3) Å^3
Z	4
Density [g·cm^{-3}]	1.6528
Temperature	100 K
TEM	FEI Tecnai 02
Radiation (wavelength)	electrons (0.0251 Å)
Precession angle	1°
Resolution range (θ)	0.07–1.15
Limiting Miller indices	*h: -28 → 28*; *k: 0 → 15*; *l: 0 → 21*
No. of independent reflections (obs/all) – kinematic (merged data)	2456/4321
R_{int} (obs/all) – kinematic	0.1551/0.1783
Redundancy	2.9
Coverage for sinθ/λ = 0.55Å^{-1}	75%
**Kinematical refinement**	
Outliers filtering condition	*|F(obs)-F(calc)|>10σ(F(obs))*
No. of filtered reflections	*11*
No. of reflections (obs/all)	2446/4310
R, *wR* (obs); *R*, *wR* (all);	0.2244/0.2873; 0.2856/0.3020
N refined param.	183
**Dynamical refinement**	
No. of reflections (obs/all) collected	41556/66950
Reflection selection criteria RSg(max)	0.5
Outliers filtering condition	*|F(obs)-F(calc)|>15σ(F(obs))*
No. of filtered reflections	*74*
No. of reflections (obs/all) (refinement)	3894/19857
R, *wR* (obs)	0.1159/0.1202
R, *wR* (all)	0.2793/0.1423
N all param./*N* struct. parameters	273/182
Effective thickness	1250 Å
Flack parameter	0.5

Table S2. positional parameters at 100K

atom	Wyck.	*x*	*y*	*z*	*Uiso [Å²]*
Si1	8b	0.5178(2)	-0.1917(3)	0.6702(2)	0.0025(2)
Si2_1	8b	0.4180(2)	-0.6756(3)	0.6252(3)	0.0025(2)
Si2_2	8b	-0.3890(2)	0.6842(3)	-0.6270(3)	0.0025(2)
Si3_1	8b	0.3214(2)	-0.1977(3)	0.6807(3)	0.0062(10)
Si3_2	8b	-0.2958(2)	0.1991(3)	-0.6895(3)	0.0062(10)
Si4_1	8b	0.4489(3)	-0.3823(3)	0.5036(3)	0.0398(12)
Si4_2	8b	-0.4244(3)	0.3834(3)	-0.5026(3)	0.0398(12)
Si5_1	8b	0.4006(2)	-0.4854(3)	0.6991(3)	0.0025(2)
Si5_2	8b	-0.4012(2)	0.4870(3)	-0.6960(2)	0.0025(2)
Si6_1	8b	0.1935(2)	-0.0112(3)	0.2159(3)	0.0025(2)
Si6_2	8b	-0.1947(2)	0.0126(3)	-0.2197(3)	0.0025(2)
Si7_1	8b	0.4156(2)	-0.2950(3)	0.6248(3)	0.0025(2)
Si7_2	8b	-0.3885(2)	0.2975(3)	-0.6179(3)	0.0025(2)
Si8	8b	0	-0.0094(3)	0.2226(2)	0.0025(2)
Si9_1	8b	0.2056(2)	-0.2215(3)	0.1764(3)	0.0025(2)
Si9_2	8b	-0.1795(2)	0.2261(3)	-0.1888(3)	0.0025(2)
Si10	8b	0.0143(2)	-0.2267(3)	0.1953(3)	0.0142(17)
O1_1	8b	0.4147(3)	-0.3409(5)	0.5589(3)	0.0627(11)
O1_2	8b	-0.3868(4)	0.3231(4)	-0.5479(2)	0.0627(11)
O2_1	8b	0.4313(4)	-0.3214(6)	0.4453(2)	0.0627(11)
O2_2	8b	-0.4126(3)	0.3347(7)	-0.4384(2)	0.0627(11)
O3_1	8b	0.3411(3)	-0.2399(6)	0.7455(3)	0.037(2)
O3_2	8b	-0.3094(3)	0.2347(6)	-0.7572(3)	0.037(2)
O4_1	8b	0.2013(3)	-0.1047(3)	0.1718(3)	0.0142(16)
O4_2	8b	-0.1872(3)	0.1106(3)	-0.1822(3)	0.0142(16)
O5_1	4a	0.4346(4)	-0.5	0.5	0.0627(11)
O5_2	4a	-0.4087(4)	0.5	-0.5	0.0627(11)
O6	8b	0.5192(4)	-0.2387(5)	0.7357(2)	0.050(3)
O7_1	8b	0.4688(3)	-0.2342(4)	0.6326(3)	0.028(2)
O7_2	8b	-0.4294(3)	0.2126(4)	-0.6313(3)	0.028(2)
O8_1	8b	0.3643(2)	-0.2250(4)	0.6289(3)	0.014(2)
O8_2	8b	-0.3302(2)	0.2623(4)	-0.6410(3)	0.014(2)
O9_1	8b	0.4091(3)	-0.5618(3)	0.6443(3)	0.051(2)
O9_2	8b	-0.3809(3)	0.5826(4)	-0.6619(3)	0.051(2)
O10	8b	0.5122(3)	-0.3711(5)	0.5172(4)	0.0627(11)
O11_1	8b	0.3662(3)	-0.7398(5)	0.6416(4)	0.039(2)
O11_2	8b	-0.3303(2)	0.7333(5)	-0.6214(3)	0.039(2)
O12_1	8b	0.4127(3)	-0.3765(3)	0.6762(3)	0.036(2)
O12_2	8b	-0.4044(3)	0.3959(4)	-0.6525(3)	0.036(2)
O13_1	8b	0.2659(2)	-0.2459(5)	0.6626(3)	0.0235(19)
O13_2	8b	-0.2336(2)	0.2164(4)	-0.6741(4)	0.0235(19)
O14_1	8b	0.3205(3)	-0.0812(3)	0.6805(3)	0.030(2)
O14_2	8b	-0.3111(3)	0.0873(3)	-0.6775(4)	0.030(2)
O15	8b	0.0138(3)	0.0741(3)	0.1728(3)	0.038(3)

O16	8b	0.0012(3)	-0.1117(2)	0.1899(3)	0.007(2)
O17_1	8b	0.0712(3)	-0.2456(4)	0.1633(3)	0.025(2)
O17_2	8b	-0.0309(3)	0.2855(4)	-0.1611(4)	0.025(2)
O18	8b	0.2412(2)	-0.0080(6)	0.2628(3)	0.089(6)
O19_1	8b	0.3386(2)	-0.4864(5)	0.7195(3)	0.027(2)
O19_2	8b	-0.3637(3)	0.4706(5)	-0.7520(3)	0.027(2)
O20_1	8b	0.43888(19)	-0.5104(6)	0.7542(3)	0.108(5)
O20_2	8b	-0.4588(3)	0.4998(6)	-0.7245(3)	0.108(5)

Table S3. X-ray powder diffraction structure refinement details at ambient temperature (~300K)

Refined structural formula	$(SiO_2)_{34}$ + 6 O (solvent)
Crystal system	orthorhombic
Space group	*C*2*ce*
a	25.0982(8) Å
b	13.7003(3) (8) Å
c	22.5228(7) Å
V	7744.6(4) Å^3
Z	4
Density [g·cm^{-3}]	1.8344
Temperature	300 K
Device	Empyrean of PANalytical
Configuration	Debye-Scherrer transmission
Radiation (wavelength)	X-ray (Cu) (λα1 = 1.540593 Å, λα2 = 1.544427 Å)
Range 2θ(°)/increment	From 3 to 80 / 0.013
**Rietveld refinement parameters**	
No. of reflections (obs/all)	1908/1908
R/wR (obs); *R/wR* (all); R_B/wR_B (obs)	0.0309/0.0370; 0.0309/0.0370; 0.1001/0.1682
N refined param.	161
Profile parameters	*Rp* = 0.0611, *wRp* = 0.0829, *GoF* = 0.1426
